# Organizational support for frontline harm reduction and systems navigation work among workers with living and lived experience: qualitative findings from British Columbia, Canada

**DOI:** 10.1186/s12954-021-00507-2

**Published:** 2021-06-05

**Authors:** A. Greer, J. A. Buxton, B. Pauly, V. Bungay

**Affiliations:** 1 School of Criminology, Simon Fraser University, 8888 University Drive, Burnaby, BC V5A 1S6 Canada; 2 School of Population and Public Health, University of British Columbia, 2206 East Mall, Vancouver, BC V6T 1Z3 Canada; 3 School of Nursing, University of Victoria, PO Box 1700 STN CSC, Victoria, BC V8W 2Y2 Canada; 4 School of Nursing, University of British Columbia, T201-2211 Wesbrook Mall, Vancouver, BC V6T 2B5 Canada

**Keywords:** Peer work, People with lived and living experience, Peer engagement, Service roles, Harm reduction work, Organizational support, Overdose prevention

## Abstract

**Background:**

The inclusion of people with lived and living experience of substance use is essential to effective and client-centered harm reduction services and strategies. The aim of this study is to critically examine and characterize peer worker roles and the definition, recognition, and support for these roles within harm reduction organizations.

**Methods:**

Fifteen interviews were conducted with peer workers—people with lived and living experience of substance use engaged in harm reduction service delivery—in British Columbia, Canada. An interpretive descriptive approach to data analysis was used to generate themes that best illustrated the roles of peer workers.

**Findings:**

Two interrelated and overarching themes are presented: (1) peer work in practice; (2) organizational support. Our findings illustrate that peer work is incredibly complex and demanding, requiring peers to be at the forefront of support within their communities while simultaneously navigating the oppressive structures within which they work. While peer workers found a high degree of purpose and meaning in their day-to-day work, their roles lacked definition within organizations, which produced feelings of ineffectiveness and being undervalued. A lack of organizational understanding and recognition of their roles was evident from unclear "peer" role titles, a lack of role communication and expectations, the representation of experiential knowledge, and a lack of role support and training.

**Conclusions:**

These findings may help harm reduction organizations understand peer work and worker roles which may inform and promote equity in future harm reduction initiatives that include people with living and lived experience of substance use.

## Introduction

North America is facing two major public health crises: the overdose epidemic and COVID-19 pandemic. In Canada, between 2016 and 2020, an estimated 17,602 people died from an opioid related overdose [1]. British Columbia has been especially affected by these dual crises—7000 of these deaths in Canada were British Columbians. In 2016, following the declaration of a public health emergency in the province, 922 deaths were attributed to drug toxicity in the province. In 2020, this number rose to 1716 deaths [2]. The opioid toxicity epidemic also overlaps with historical epidemics of HIV and hepatitis C transmission [3]. Collectively, these overlapping epidemics have required a multitude of public health and policy responses including the increase of harm reduction services and strategies.

Promoting the inclusion of people with lived and living experience (PWLLE) of substance use in public health initiatives is central to harm reduction policies and practices [4–6]. There is a long history of grassroots organizing of PWLLE in the harm reduction field which laid the foundation for such inclusion, including grassroots self-organizing among PWLLE for harm reduction and human rights during HIV crises of the 1970–1990s [7–9]. For instance, in 1997 in British Columbia, the Vancouver Area Network of Drug Users (VANDU) formed in response to an HIV crisis among local people who inject drugs, focusing on “bring[ing] the ‘voice of users’ into mainstream political discourse” [10, p. 63]. For over two decades, VANDU (among other self-organizing groups of PWUD) has provided peer support, education, advocacy, and harm reduction programming [5, 10] and has been involved in a range of research [11]. Today, self-organizing groups of PWUD continue to be at the forefront of public health efforts to ensure they are included in decisions and initiatives that affect their community [7]. Today in British Columbia, the inclusion of PWLLE is promoted as a crucial part of overdose response efforts by the Government of British Columbia [12] and is a priority item for several provincial overdose task groups [13, 14].

A growing body of North American literature demonstrates the positive impacts of engaging peer workers in policies, programs, the community, and work settings [15]. Peer work of all types provides employment and income for PWLLE who have historically been excluded from the labor market [11, 16]. Studies show peer-based harm reduction services are preferred by PWLLE and increase the uptake of initiatives [17, 18]. Peer-based service work is valued due to peer workers’ ability to build relationships and trust, challenge power hierarchies, and increase the relevance of programs and services [18]. Benefits to service workers themselves include a sense of inclusion, connection, empowerment, and agency [19, 20].

Despite the benefits of peer-based service work, there is consistent evidence of challenges to hiring peers within organizations [21]. Peer workers face a disproportionate amount of pressure, burden, and trauma in their work, especially in overdose prevention contexts [17, 20, 22]. Studies also show that peer workers receive minimal social and emotional supports [17, 20–22], which may be partially attributable to their casual work arrangements [23]. Questions are being raised about the sustainability or potential burnout of the peer workforce, especially in the context of the overdose crisis in North America [21, 23, 24]. More research is needed that examines the role of organizations in providing support to peer workers in this context.

Peer-based work makes up an umbrella under which there are a variety of peer roles and responsibilities. Marshall et al. [25] review the literature and categorize five distinct peer roles in harm reduction settings, including education (e.g., creating educational materials), direct service (e.g., distributing injection supplies), support and counselling (e.g., facilitating groups), research assistance (e.g., collecting data), and advisory committee participation (e.g., policy input). The review and others note that peer roles serve a purpose but individuals have a low degree of involvement in decision-making [25–28]. These observations suggest that peer roles may be influenced by power gradients that are organized and operate within hierarchical organizations. To date, peer workers’ roles have been primarily discussed in terms of the degree of involvement in services and strategies [25–27], rather than the utility or effectiveness of peer workers’ roles and responsibilities within organizations—an examination of these factors is still needed.

Although researchers have highlighted the benefits and challenges of peer work, there has been limited exploration of the specific work, organizational context, and operation of peer roles. There is a need to examine the influence of the organization and employers in enabling and supporting this work. The aim of this study is to critically examine and characterize peer worker roles, along with the definition, recognition, and support for these roles within harm reduction organizations. We qualitatively examine the experiences and roles of peer workers who provide support, services, and outreach to others, with a specific focus on workers doing frontline harm reduction work and systems navigation work in British Columbia, Canada. We unpack the ways in which these roles operate and are utilized by organizations, and the impact on worker agency and recognition.

## Methods

In this study, we employed a qualitative research design guided by interpretive description, a qualitative methodology that requires data analysis to go beyond description and emphasize interpretation and application [29]. Interpretive descriptive methodology utilizes techniques to discover potential associations, relationships, and patterns described by looking at underlying meanings to illuminate what is happening, while considering the real-world context [29]. This methodology allowed for the use of a range of qualitative methods which complemented and facilitated an intentional and purposeful inquiry.

In 2017–2018, we conducted fifteen semi-structured qualitative interviews with PWLLE from across British Columbia. Participant inclusion criteria included being over the age of 16 years and identifying as a peer worker in the past twelve months. A peer worker was defined as a person with past or present substance use experience who uses that experience to inform their professional work. Peer work was defined as both formally or informally arranged work as well as paid or unpaid efforts related to harm reduction services for PWLLE. Participant recruitment for this study included word-of-mouth through peer networks, advertisements at harm reduction agencies, and snowball sampling through participants. Five interviews occurred over the telephone to include the perspectives of people living in rural and remote areas. Purposeful sampling paired with concurrent data collection and analysis drove the recruitment, identifying and targeting participants who offered a variety perspectives and experiences of peer work [30]. For example, we ensured participants with perspectives of peer workers in both full and part-time or casual work were included, as well as those with a long work and short work tenure. We also ensured participants were recruited across various jurisdictions and organizational settings.

Prior to the interviews, participants were provided with a study information sheet and consent form. The consent form was reviewed again before the interview started and any questions or concerns were addressed. All participants gave informed consent before participating. The lead author conducted the interviews as part of her doctoral research. All authors worked with peer workers on various projects across the province prior to the study and knew some of the participants in the project prior to their participation. The interviewer was mindful of these relationships, the line of questioning, and interpretation of the data. She also emphasized that the interview would not impact the relationship and that confidentiality would be maintained. They were also encouraged to speak honestly and openly.

The final sample comprised of fifteen participants. After thirteen interviews, we were confident in the richness of the data collected in that it illustrated the complexities of peer workers’ experiences and felt complete but conducted another two interviews to reinforce the findings and conclusion that further gathering of data would not create a significantly deeper understanding. The fifteen interviews provided a sufficient richness and depth in the data, as was evident in the patterns and themes that emerged during the concurrent sampling and analysis [30].

Interviews were 45–90 min in length. Most were conducted in person, although five were conducted over telephone due to geographical constraints. A semi-structured question guide informed the interviews. The questions were based on and informed by previous knowledge of peer work, peer work literature, insights from peer workers, and the ongoing interviews themselves. The questions focused on peer roles, responsibilities and duties, employment and hiring experiences, the pay process, the work context, and recommendations. Each participant received a $20 CAD cash honorarium, refreshments, and transportation reimbursement. Transcription of audio recordings occurred within one week of the interview. The data were deidentified of any personal information (i.e., names, places, agencies). Given the small community of peer workers in the province, no identifying information is provided with the quotes.

All data were imported into and organized in NVivo [31], a qualitative data management program. Data analysis was nonlinear, collaborative, and iterative throughout data collection and thereafter. The lead author (AG) took the lead on coding and met regularly with co-authors to discuss the emerging themes. Coding was an evolving process that facilitated understanding and meaning as relationships and patterns emerged from the data [32]. For the findings presented in this paper, the analysis focused on the descriptions and perceptions of peer workers about their roles in harm reduction work, the utility of these roles, organizational and operational constraints, and recognition of peer workers’ efforts.

In line with interpretive description, the aim of the analysis is to both identify and infer different unseen or unspoken factors that explained how or why participants were describing and perceiving peer work and their roles in particular ways (or not) [29]. We paid particular attention to the structural and/or organizational mechanisms and factors that were tied to peer workers’ experiences. How organizations put peer worker roles into operation was often articulated in descriptions of their day-to-day work and the challenges workers faced. In this paper, we present the roles of peer workers in harm reduction settings and how these roles are defined, supported, and recognized in organizations. Other study findings, including an examination of peer work conditions, are presented elsewhere [23].

The "trustworthiness" of interpretive research is in making the complexities of study procedures visible with an openness [29]—an openness embraced by the research team and evident in the many strategies employed to enhance the validity and reliability of the research. We practiced reflexivity, including making interpretive claims explicit and acknowledging researcher power, privilege, and positioning on the research. The research team also ensured that the analysis was collaborative by using multiple perspectives and interpretations and triangulating these insights. We also validated the findings by bringing the findings back to the community [33, 34]. Both during and after concurrent data collection and analysis, we had conversations with PWLLE and others about the emerging findings. These conversations added credibility to the findings by having purposeful, regular, yet informal discussions with peer and non-peer workers (i.e., employers or supervisors) about what was emerging from the data. These conversations added value to the analysis by verifying or challenging the interpretations, while also validating the perspectives and experiences of the community. Some people provided feedback that they enjoyed or valued these conversations.

This study and its procedures were approved by the University of British Columbia Research Ethics Board (#H17-02039).

## Findings

### Description of the sample and work context

The participants’ demographics are provided in Table 1. The median age of participants was 45 years (range 27–60 years) and over half of participants identified as female (*n* = 8). Participants self-identified primarily as Caucasian (*n* = 10; 67%). Participants were from diverse regions throughout the province. The length of time engaging in peer work ranged from three months to over 20 years, with a median of 7 years (Table 1).

**Table 1:** Characteristics of participants (*N* = 15)

	*n*	%
*Age group, in years*		
25–34	2	13.3
35–44	5	33.3
45–54	6	40.0
55+	2	13.3
Median	44	
Range	27–60	
*Gender*		
Female	8	53.4
Male	7	46.6
*Ethnicity*		
Caucasian	10	66.7
Visual minority	5	33.3
*Time doing peer work, in years*		
< 2 years	5	33.3
2–5 years	1	6.7
6–9 years	5	33.3
10+ years	4	26.7
Median	7	
Range	0.25–30	
*Substance use history*		
Within past 12 months	8	53.4
More than 12 months prior	7	46.6

Participants’ descriptions and experiences provided insight into peer work contexts. Types of organizations included peer-based organizations, health authorities, and non-profit organizations that provided harm reduction services (i.e., overdose prevention sites, needle, and syringe programs). The number of work opportunities were limited, and many were short term or casual; therefore, the majority of participants regularly worked across multiple organizations. Just four participants were formally employed and two of these held full-time employment. Generally, participants’ experiences were not specific to one organization but often spoke to their roles in multiple organizations. Participants rarely attributed an experience to the organization itself or stated differences between organizations, such as roles in peer-based organizations versus among health authorities or not-for-profit organizations.

Below, we organize our findings into two overarching and interrelated themes: peer work in practice and organizational support for peer work. The first theme, *"Peer work in practice,’"* provides a description and characterization of two prominent types of work and the roles they take on: frontline harm reduction work and systems navigation work. In the second theme, *organizational support for peer work*, we examine how peer worker roles are defined, utilized, recognized, and supported through four subthemes: the meaning of the "peer" role title; communicating role expectations; utilizing experiential knowledge; and offering role support and training. These themes and subthemes are listed in Table 2.

1. **Peer work in practice**

Peer worker roles are characterized below according to two prominent types of work being done within organizations. First, *frontline harm reduction work* included activities such as distributing harm reduction supplies, overdose response, outreach, peer education, and providing clients with meals and other services. Second, *system navigation work* included relatively more informal duties such as assisting others filling out paperwork, navigating governmental policies and programs, and accompanying other PWLLE to appointments and meetings. Below, we critically examine the key features and nature of roles in these lines of work.

**Table 2:** Themes and Subthemes related to peer work and worker roles in harm reduction organizations

1. Peer work in practice	Frontline harm reduction work System navigation work
2. Organizational support for peer work	The "peer" role title Communicating role expectations Utilizing experiential knowledge Offering role support and training

#### Frontline harm reduction work

Peer worker roles in frontline harm reduction work included responding to overdose, street outreach, peer health education, peer emotional support and groups, distributing harm reduction supplies, and providing meals. Much of this frontline work occurred in the context of overdose prevention and response settings. Frontline harm reduction roles were characterized by two main features: incessant emotional demands and their connection to the community. First, frontline harm reduction work required peer workers to navigate numerous emotionally demanding tasks, such as helping overdose victims, working in front of a crowd, interacting with police officers, performing medical procedures, and providing emotional support to others. Participants used vivid language to describe this work, and some cried while they spoke. Some participants recognized the emotional toll of the work, including regularly witnessing overdoses: “*if you’re boiling someone an egg at 8:00 and by 9:00 the next morning you find out they’re dead, you know, it’s very jarring. But you can’t stop what you’re doing*.” The emotional demands of their work were paired with a sense of urgency to be involved. Peer workers were determined to continue working despite the emotional challenges of frontline harm reduction work.

In addition to the emotional demands of service roles, frontline harm reduction work was profoundly and pervasively characterized by peer workers’ connection to the community. Peer workers worked directly with their own personal community and found their work benefited from their experience and connection to it: *“It’s just kind of who I am, it happens every day”*. Participants also talked about the benefit of their connection to the community in their ability to provide services to networks of hard-to-reach community members. Peer workers could reach people that other service providers could or would not. However, peer workers’ connection to the community presented an inseparable overlap between their professional and personal lives which complicated frontline harm reduction work and the demands they faced. The embeddedness in the community, along with the sense of responsibility and urgency to meet community service needs, placed undue work demands peer workers. One person explained:

Like, hey, aren’t you the person that I saw this morning? So, you do end up volunteering your time back to the community because once you’ve been doing a lowly paid stipend job like that for a while, all the people know you. And they expect you to be there. They don’t know the difference between a volunteer and an employee if you’re living on the street. They just know you come by every day at 7:30.

It was particularly challenging for peer workers to disengage from their service roles, given that the community where they live was also their workplace. Some participants contrasted how these roles differed from non-peer workers. “*You realize—hey, this is real stuff, you know, the bureaucrats go home at the end of the day. But I stayed”*. Again, as peer workers personal community were the people they provided services to, some felt their work was incessant; 24/7.

#### System navigation work

Several participants described a unique role in their community where they helped PWLLE navigate and confront different systems, policies, and procedures, such as social assistance and housing programs. Some participants referred to this role as “*systems navigation*” or “*office outreach”*—a role that peer workers did indoors, helping others traverse, understand and overcome systemic inequities including homelessness, and poverty. Duties and tasks included assisting PWLLE with applications, opening bank accounts, providing support at healthcare or social assistance appointments, and accessing housing. Some participants described this role as facing “*political red tape*,” working within the “*bureaucratic system*,” helping with paperwork or the “*administrative nightmare”*, and “*helping people understand, you know, who holds power, how to access it, strategizing, stuff like that*.”

Participants described the utility of their own lived experience of inequity as a form of expertise that they used in system navigation work. Participants emphasized using this lived experience in their roles, as highlighted by one participant:

I was an injection drug user. I was a sex worker… I had gone through, in my conflict with the law. So I brought all of that and, you know, system navigation skills to my work. And then a whole lot of empathy and compassion and passion.

Participants spoke to the expertise gained through their lived experiences and value it added, such as compassion for others facing high barriers in various systems. Some emphasized about their knowledge and understanding of “*bureaucratic language*” as a skill used to support others:

There’s a certain amount of bureaucratic language that most people just don’t understand. And there’s a lot of, like, I’m not sure the proper term for it, but I think it’s almost like a defense mechanism for bureaucratic people to start bringing out the big words when they’re not sure how to respond. Which creates kind of a language barrier, I guess, and it’s condescending, and people can sense that. But they don’t have the vocabulary to respond to it. So, it’s stressful for some people.

Peer workers translated language and information in a way that both PWLLE and the bureaucracy understood.

In peer workers description of their work in practice, it was particularly evident that much of their efforts were not recognized, both formally and informally, by their employers or harm reduction organizations. Peer workers connection to the community placed inescapable expectations on them to be available any time or place. They felt that others relied on the embeddedness of peer workers in the community, and some workers found it difficult to decline requests to fulfill the service needs in the community. However, participants explained that, at times, peer workers connection to the community could be leveraged for organizational benefit at the expense of peer workers’. One participant explained:

I have, like, an underground network of people that contact me… because I can go get her. I’m not a professional. My job isn’t on the line. It doesn’t matter, I’m not breaching any propriety agreement or code of conduct …This is stuff that people do because they genuinely care about the people that they’re taking care of… [but] there’s logistics involved. I have to stop whatever I’m doing to go respond to a crisis call, and I don’t get paid for any of this. So, I can only do so much.

The work of providing services to perceived hard-to-reach communities by systems was almost always unsupported or unrecognized in terms of the time, effort, and emotional demands they experienced. Despite harm reduction organizations benefiting from peer workers positioning, this type of frontline service work was universally not paid or formally recognized by organizations.

However, most system navigator roles were not formally recognized by organizations and they lacked compensation: “*I do this work for free.”* Furthermore, they were not offered role or social supports. Despite the lack of value provided by organizations, peer workers felt valued. Knowing the difference that systems navigation work provided to other PWLLE was motivating. Helping people, demonstrating leadership, and impacting the community produced a deep sense of pride. Again, however, peer workers motivation could be leveraged or reinforce the invisibility of important systems navigation work.

2. **Organizational support for peer work**

In this theme, we examine the definition, utility, recognition, and support in peer work which speaks to the understanding for peer work in organizations. Below, we examine organizational support for peer work and their roles through the lens of workers themselves. Four interrelated subthemes are presented: (1) the "peer" role title; (2) communicating role expectations; (3) utilizing experiential knowledge; and (4) role support and training.

#### The "peer" role title

Role titles indicate workers’ positions, responsibilities, and scope of work within organizations. For PWLLE, having the title of "peer" was common in harm reduction work. Participants were asked about this title and other role titles they were given, if any. Participants regularly lacked a role or job title and, because of this, were often unclear of their role responsibilities altogether. Many participants responded by stating they were “*just a ‘peer’*” without defining what the "peer" title meant or how it related to their role or responsibilities. Some participants had relatively more description in their job titles, such as “peer coordinator” or “peer outreach worker,” compared to those who simply attended meetings or informed on policies under a blanket "peer" role title.

Role titles for peer workers were important for developing legitimacy and occupational identity. For instance, one participant explained: “*it’s [the word ‘peer’ is] important because it’s current in discussions”*, speaking to legitimizing and promoting the representation of PWLLE in harm reduction organizations. Similarly, others explained that role titles provided an “*understanding [of] peer work itself*” in a context where their roles were “*not recognized universally*” by employers or coworkers. Creating a well-defined occupational identity had the potential to increase the recognition and integration of the peer role into organizations. As one participant explained: *“[other staff] might be more trained in a society way and people can respect a ‘scientist’ and a ‘nurse’*—*and so we live in a funny, like, in between”*. Unlike other occupations which are clearly defined, like a nurse, teacher, or accountant, there was no universal knowledge about what activities occur under the "peer" title.

In contrast to those who saw the "peer" role title as providing legitimacy and an occupational identity, others alluded to the disadvantages of it. Some expressed that the title of "peer" introduced stigma given its connection to substance use. One person said:

I don’t know if you’re interested, it’s not semantics, but it’s saying persons with lived experience rather than 'peers' because there’s becoming a thing in the neighborhood where 'peer' is starting to feel like a put down in a weird way.

Participants spoke to the disadvantages such as pay inequity and labor discrimination due to being labelled as a "peer" worker. They explained that peer workers were paid, supported and organized different from non-peer workers. In talking about the "peer" title, one participant stated: “*the way it gets paid… there’s us [without the peer title] and then there’s people who are working for stipends and stuff like that”*. This title limited some workers to informal or temporary work arrangements and the low and inconsistent pay conditions associated.

#### Communicating role expectations

In addition to vague role titles, participants regularly did not have details about their roles or responsibilities. Participants commented on lacking key information needed in their roles about programs or issues at hand, role expectations, the degree of involvement and decision-making, and if or how their efforts were utilized or recognized. Some explained the importance of providing “*enough [information] that people were secure in sharing or knowing what to share and what not to share*.” Communicating these expectations were important for empowering workers to contribute.

In contrast, a lack of communication about their role and expectations was disempowering. Defining role expectations early on in engagement with employers was especially important in setting peer workers up for success. However, when asked in the interviews, many participants could not specify a conversation with employers about what their roles or responsibilities. One participant’s quote emphasizes the implications of setting expectations though clear communication about their roles:

I wish that I would have more time to have gone over the stuff… Because then somebody kind of came in behind me and took over… I needed to know what kind of information they were going to tell me. I needed to know what they were going to, kind of things that they were going to be asking so that I could have been more prepared… it was a last-minute kind of thing.

The example coneys that workers were unsure how to use their lived experience when they received no information about what the work entailed. As a result, the worker felt unprepared and unvalued in their role.

The lack of communication about role expectations indicated that employers’ themselves did not clearly understand peer work. For instance, one participant who had no pre-defined role responsibilities was asked by their employer to define the role themselves. They said: “*With this position… they’re allowing me to create it as I go along. So they don’t have a whole lot of ‘this is what we expect.’”* Although this participant could exercise agency and autonomy in their role, they were simultaneously unaware of how to meet any unstated role expectations and felt a lack of recognition for their role. Considering that workers efforts were often not recognized by employers, workers were left vulnerable to perceptions of underperformance and potential job loss if they did not meet unstated role expectations.

Furthermore, without clear role expectations, peer workers simply guessed what their employers expected. However, such inadequate or unclear role expectations produced confusion for workers whose roles seemed to encompass any and all activities. One participant stated: “*[my role is] eclectic. There’s a lot of different things I end up doing. And it could go off in all kinds of different directions”*. Similarly, a woman explained: *“always something I can make up … answer the phones, clean up, whatever it is*.” The inadequate information peer workers received regarding roles created endless possibilities of what peer workers’ role duties entailed.

#### Utilizing experiential knowledge

Participants spoke about the ways peer workers’ skills and experiential knowledge was utilized and represented in harm reduction organizations. Overall, the representation lived and living experiences of peer workers and utility of their unique knowledge appeared to be misunderstood and underutilized in organizations. This lack of understanding and utility was evident both in the misrepresentation of experiential knowledge in peer worker roles and underutilization of their unique knowledge. Participants explained that PWLLE were often engaged under a broad stroke of "substance use" rather than any specific knowledge or expertise they may have. This was particularly evident where their experiential knowledge did not relate to their role. As one participant described:

Sometimes I think [employers] just don’t know enough and they’re trying to do the right thing…we’re going to hire the first person that identifies as Aboriginal. You know, like, go find me some Indians... Hey, have you done sex work? Can you come and I got a job for you! That’s not okay…a peer can be defined in many different ways. It can. And there is a spectrum, a continuum, right, you have a peer who’s, you know, completely still street involved, using drugs… someone who’s specific, like, say, sex work, you have someone specific to HIV, it is really, really diverse and multidimensional.

As in this example, the lack of attention paid by employers to ensuring their lived experience or expertise was compatible with the issue at hand showed their lack of understanding for the diverse experiences of PWLLE. It also indicated that employers did not fully recognize the utility of the knowledge gained from their experiences when the issue at hand was incongruous with their personal lived or living experience. A person whose lived experience was with stimulants explained:

I haven’t really shared much yet… Because I don’t use opiates and I don’t use, I’ve never been in the position of helping overdoses or saving someone or any of that stuff… So I don’t feel like I’m on the same, I haven’t gone to healthcare because I was overdosing and been treated like crap by the doctors. I’m a crackhead... I don’t feel like it’s the same [as opioid use].

As this quote indicates, participants with living stimulant use experience expressed an inadequacy working in roles requiring an opioid use knowledge. Treating substance use as a heterogeneous experience for certain types of work was disservice as it was difficult for them to meaningfully contribute and feel effective or utilized in their work.

Participants were also delegated to jobs unrelated to this expertise. Some participants stated that they were hired as "peer" workers but were assigned to tasks such as cleaning, cooking or answering phones. These participants expressed a desire for their skills to be more meaningfully utilized. Both where peer workers’ experiential knowledge was not used and where it was incongruous with the issue at hand, their roles appeared to be more symbolic or tokenistic than intentional or meaningful in organizations.

#### Offering role support and training

Role support, including organizations providing the means to workers to do their work effectively such as training, resources, education, funding, and tools, was consistently lacking in peer work. Participants emphasized the need to provide training for peer workers. “*I wish for someone like me, that I could be offered to learn to do better in what I do*… *I wish I could do a little bit more and learn a little bit more*.” This desire was linked to the opportunity to perform effectively, and disadvantage when they were not provided with this training. For some participants, the lack of training and support indicated a lack of understanding for peer work within organizations. For example:

There’s not a whole lot of education around what it takes to support a peer… there’s a lot more to it than just going “here’s a job”… people should really if we want to, you know, show value and build capacity, I know lots of co-workers that don’t come from lived experience that get tons of training and they have money provided for it... I call it capacity building, but they call it something else... everybody should be treated the same kind of thing, right, but some people will have unique needs as far as education.

This quote underscores the inequity peer workers experience within organizations when peer work is unsupported. When participants are underprepared or when their capacity is not supported, they experience what this participant referred to as “*extreme tokenism.”* Given the lack of role supports, peer workers reported feelings of inadequacy, disappointment and uncertainty in their roles, as well as a sense of being undervalued by the organization itself.

Despite the lack of role support, workers took it upon themselves to adapt and build capacity. “*I self-taught myself everything that I know including how to work at [organization].*” Peer workers demonstrated their resilience, adaptability and tenacity in their roles, finding the tools, and educating themselves even when organizational role support was not accessible.

The importance of role support in peer work was reinforced among participants who actually received training or other supports from their employers. For example, some participants were provided with computers in their roles and offered computer training. The sense of pride and adequacy that comes from such support was evident among one participant who talked about their computer training: “*learning how to turn it on and not be scared of it, get the feel. And they’re so excited, and I’m so excited*!” This investment from employers conveyed a sense of value and recognition for peer work. Others talked about the empowering aspects to role support.

[It’s a] power sharing model where you pull somebody in and you help build them up and you give them the tools and not have expectations. Especially if you know you’re hiring peers that don’t have degrees or social work ethics [laughs] these kinds of things that they all expect that there needs to be, you know, a little bit of training around that and support.

Acquiring the necessary skills and tools to effectively perform created a sense of empowerment in their roles. However, not all training provided was sufficient. Some workers experienced a mismatch between the training provided and their role—again reinforcing a lack of organizational understanding of peer roles. Participants expressed more training was needed to educate employers about “*what peer engagement is*,” as well as how to use and apply peer workers expertise and lived experience within organizations.

## Discussion

This study provides rich insights into the practice and organizational understandings of peer work including the ways peer work is defined, understood, and utilized in harm reduction organizations. Peer workers lack role clarity, expectations and support; this lack of clarity impeded the recognition, utility, and effectiveness of workers’ efforts. Despite trying hard to do good work while remaining flexible and responsive, the lack of clarity for organizations sets up for failures rather than success. Peer workers faced job insecurity and risked not meeting unstated organizational expectations or appearing ineffective. Our findings suggest that peer worker roles and the inclusion of PWLLE may not be fully understood or supported by organizations. This study provides valuable knowledge about the ways the unique knowledge among PWLLE can be utilized and supported in harm reduction work.

Being denied the means through which peer workers could effectively do their work was a major factor that limited the recognition and utility of their efforts. This finding suggests that inadequate training, a lack of role support, and tokenistic inclusion may be continuing in harm reduction settings [27, 25]. Participants were positioned in roles which did not utilize their unique skills or expertise, suggesting that employers may not fully understand the value of peer workers’ roles or how to utilize them within their organization. This finding provides strong support for other studies that suggest employers and non-peer co-workers may not understand what peer workers do or the value they add [26, 35, 36].

Our findings highlight the ways that participation gets carried out in harm reduction work and demonstrate the realities of how the organization of peer work is experienced by workers themselves. The intention of inclusion and participation indicate empowering principles [4]. As written in the Nothing About Us Without Us guidelines [5], “within organizations…working with a person who uses drugs can help people overcome their prejudices and change their perceptions about people who use drugs” (p. 32). However, the lack of role clarity and expectations impedes peer workers to perform effectively and have their efforts recognized. Despite seemingly good intentions, peer workers were not enabled to effectively perform, contribute or act. Role operationalization is foundational for how participatory processes proceed or are accomplished. Our findings echo others who suggest that if workers are “denied the means through which they can participate… social inclusion as ends becomes purely aspirational” [37, p. 201]. For instance, Belle-Isle [26] shows inconsistencies in role support, hiring and paying peer workers and their negative impact on worker agency. Our findings indicate a perpetual a lack of attention to the implementation of peer-based work *in practice*, underscoring the taken-for-granted nature of participation and inequity that is often overlooked both in harm reduction settings [27, 38].

The goal of "peer engagement" is to empower and utilize peoples’ lived and living experiences of substance use and their unique ability to connect with the community to promote the relevance, accessibility and equity in harm reduction initiatives [16]. PWLLE voices have been centered as a result of a long history of grassroots organizing and activism in the harm reduction community and beyond. In contrast to grassroots, user-led advocacy for the rights of PWUD, peer engagement today can be seen taking shape as a top-down, institutionally led and motivated initiative [16]. Researchers and scholars have noted how the involvement of PWUD appears to have evolved from primarily self-organizing PWUD harm reduction efforts, to now engaging peer workers more operationally with mainstream public health institutions [39–44]—a distinction that Albert [45] has called the difference “between what we can crudely call "bottom-up" or drug user-led, and "top-down" or professional approaches to harm reduction” (p. 7). The current study offers important new insights into the ways that PWLLE efforts and roles are shaped and constrained by large institutions under the pretense of "peer engagement," but suggest that its aims may be superficially inferred or framed by employers who benefit from yet severely underrecognize and underutilize peer worker roles [46]. These findings therefore raise questions about the appropriation and ongoing institutionalization of peer workers within harm reduction organizations. This study may therefore be of vital importance for future initiatives in the organization of peer work systems which may be susceptible to perpetuating similar systems of inequity that risk undermining the goals of "peer engagement" altogether and diminish the value of the role of peer worker advocacy and activism.

Our study also uniquely begins to problematize and raise questions as to how the word "peer" is used by organizations. Institutions continue to reproduce the word "peer" in harm reduction settings to identify and describe experiential workers [22, 27, 47, 48]. Our own work has contributed to these labels [16, 27]. Conversely, the term "peer" labels workers by whether or not they have a substance use history, thus, systematically differentiating PWLLE from other workers. In the past, PWLLE has problematized this term:

The use of the word 'peer' is also increasingly being used to mean 'person with lived experience' in the context of including [PWLLE] in research, service delivery, and policy settings. While some of us welcome the use of the word 'peer' and have embraced it as a word that recognizes and acknowledges our lived experience, there are situations where identifying people as 'peers' can be problematic. [7, p. 1]

Although the term has come to promote jobs for PWLLE in harm reduction settings, in some contexts this term may also produce unintended consequences, such as discrimination, as it specifically labels and differentiates workers who are engaged for their lived experience of substance use. Furthermore, this term may be appropriated by institutions rather than being defined, used, and reproduced by PWLLE themselves.

Given the potential for participatory practices to challenge or reinforce negative discourses regarding PWLLE in the labor market, the finding that peer worker efforts were often unrecognized was concerning. A lack of understanding of what peer workers do among employers and coworkers may perpetuate negative stereotypes of PWLLE as a workforce and unacceptance or undervaluing of peer work [26, 35, 36, 49]. Without a true understanding of peer work, its utility and its value, peer workers risk being viewed as ineffective or unvalued, particularly if they continue to be unsupported. Harm reduction initiatives that include PWLLE as peer workers must continue to push for organizational support and resources to support this work [27].

Previous scholars demonstrate similar consequences from inconsistent and informal work arrangements. For instance, Standing [50] suggests that informal workers are often compelled to account for their time and efforts to challenge "lazy" discourses about their social identities, which can be difficult in work arrangements that do not enable agency, effectiveness, or opportunity. Other research shows that peer work is precarious, characterized by insecurity, low wages and a lack of social benefits [23]. Perpetuating such work arrangements may continue without the formal recognition or understanding of peer worker roles by employers. Findings in the current study suggest that organizations should acknowledge or enable peer workers’ efforts, aspirations, and activities, to promote the value of peer work because of a lack of recognition of their efforts or skills.

The current study adds to a small but growing body of literature that emphasize the structural forces that shape PWLLE’s labor market and workplace experiences [51, 52]. In examining the factors that contributed to role utility, support, and recognition, our study underscores the structural drivers of the realities of peer work, thus challenging individualistic notions of worker agency and outcomes. Although participants demonstrated worker agency in the many ways that they were aspirational, industrious, productive, and resistant, they were also consistently constrained from working effectively given the lack of role clarity, expectations and support. This study advances the current literature in underscoring the importance of peer worker role recognition and understandings of these roles to promote equity and meaningful peer engagement in harm reduction moving forward.

The apparent underutilization and lack of formal recognition for peer worker efforts suggest a lack of organizational understanding and commitment to working with PWLLE and there are clear areas of improvement in terms of organization and support of peer work. Findings suggest that improvements need to be made in communication, role expectations, role definition and clear job titles. One thing that may promote the commitment, support, and understanding of peer work is establishing a forward-facing organizational commitment for the equitably inclusion of PWLLE that may systemically enhance the value and legitimacy of peer work and produce more significant opportunities for inclusion. As well, education is needed which speaks to peer workers’ roles, including the added benefit of their skills and knowledge and its fit organizationally can enhance organizational understanding for peer roles and thus, promote role support, legitimacy, and recognition. In addition to education about peer workers’ roles, there is an opportunity to educate people within organizations about the structural determinants of work equity and their link to poverty, social inclusion, and wellness. Bringing awareness to the root causes of inequities can make them visible institutionally and may promote the opportunity to redress systems that may inadvertently reproduce inequities through the structural organization of peer work. More research is needed which considers any institutional barriers or constraints that may inhibit these important areas of improvement.

To date, few studies have taken an organizational or operational lens to peer work in harm reduction contexts. Outside the harm reduction literature, there is evidence and emphasis on the importance of role operationalization among workers with other types of lived or living experience of social marginalization. Specifically, in the field of mental health, several studies across a variety of settings echo our findings that suggest unclear and ambiguous roles and operationalization of these roles creates frustration and confusion among peer workers [53–56], as well as insecurity about demonstrating their potential and value [57]. Unclear roles are also linked to feelings of exclusion and unacceptance in mental health workplaces [53] as well as difficulty for staff and peer workers to work together [54]. Similar to our findings, mental health peer workers resort to training themselves and taking on more responsibility which impacts their capacity to effectively perform and support others [53]. Gates and Akabas [54] recommend better defined roles as well as clear structures, policies and procedures that could facilitate greater role support and expectations.

A strength of this study was employing purposive sampling which facilitated a diversity of participants in terms of age, gender, ethnicity, and roles. However, peer work in British Columbia is a close and tight knit community which occurs within a limited number of organizations. Participants often attended the same meetings or worked in the same settings. While these perspectives were reinforcing and triangulated insights into the same context, it was also a sampling limitation. Participants’ experiences and roles may reflect a common discourse and limited number of organizational processes rather than the diversity across communities and settings. However, our findings provide important insights into the organizational and operational processes that can be considered in future initiatives. Some studies suggest that organizational practices and employment may differ between formal healthcare systems versus community-based organizations which may be related to cultural differences [22, 36, 58]. In our study, these differences were not apparent although may be a limitation of the sample who were recruited as peer workers in harm reduction settings. It may also be a limitation of the location of the study. British Columbia is a unique context in terms of drug policy and harm reduction, the opioid overdose crisis, and diversity in regions across the province. This context likely shaped participants’ experiences and our interpretations of them. Furthermore, since collecting data, the opioid crisis has not abated. The peer workforce is evolving, as are the roles and organizational environments and structures they work within. As such, findings may be different if data were collected today. Given the evolving context and work conditions amid the ongoing overdose crisis and COVID-19 pandemic, more research on the organization and support of peer worker roles, as well as the roles they take on in these dual crises, is needed.

## Conclusion

In conclusion, the findings of this study underscore the importance of organization understanding, recognition and support for peer work in harm reduction. Moving forward, there are a range of roles that peer workers will continue to take on within harm reduction services and strategies—particularly in the context of the overdose crisis. There is room for improvement with regards to organizing the operation of peer work, such as building organizational understanding of peer roles, promoting communication and training, and building the capacity of organizations, to legitimize and support this important work. In doing so, peer workers’ efforts and aspirations, skills and expertise may be truly recognized, valued, and integrated.[46]

## Data Availability

The datasets generated and/or analysed during the current study are not publicly available due to confidentiality to preserve the anonymity of participants.

## Abbreviation

VANDU: Vancouver area network of drug users
PWLLE: People with lived and living experience
